# Evidence for infection in intervertebral disc degeneration: a systematic review

**DOI:** 10.1007/s00586-021-07062-1

**Published:** 2021-12-04

**Authors:** Isabelle Granville Smith, Nathan P. Danckert, Maxim B. Freidin, Philippa Wells, Julian R. Marchesi, Frances M. K. Williams

**Affiliations:** 1 Department of Twin Research and Genetic Epidemiology, King’s College London, 3rd and 4th Floor, Block D, South Wing, St. Thomas’ Hospital, Westminster Bridge Rd., London, SE1 7EH UK; 2 Department of Metabolism, Digestion and Reproduction, Imperial College, London, UK

**Keywords:** Bacteria, Degenerate disc, Intervertebral disc, Microbe, Modic change

## Abstract

**Purpose:**

Back pain is a major problem worldwide and is linked to intervertebral disc degeneration and Modic change. Several studies report growth of bacteria following extraction of degenerate discs at spine surgery. A pathophysiological role for infection in back pain has been proposed.

**Method:**

We conducted a PRISMA systematic review. MEDLINE, PubMed, Scopus and Web of Science were searched with the terms Modic change, intervertebral dis*, bacteria, microb*, and infect*. Date limits of 2001–2021 were set. Human studies investigating the role of bacteria in disc degeneration or Modic change in vertebrae were included.

**Results:**

Thirty-six articles from 34 research investigations relating to bacteria in human degenerate discs were found. *Cutibacterium acnes* was identified in pathological disc material. A ‘candidate bacterium’ approach has been repeatedly adopted which may have biased results to find species a priori, with disc microbial evidence heavily weighted to find *C. acnes.*

**Conclusion:**

Evidence to date implicates *C. acnes* identified through culture, microscopy and sequencing, with some suggestion of diverse bacterial colonisation in the disc. This review found studies which used culture methods and conventional PCR for bacterial detection.

Further agnostic investigation using newer methods should be undertaken.

**Supplementary Information:**

The online version contains supplementary material available at 10.1007/s00586-021-07062-1.

## Introduction: back pain and Modic change

Back pain, particularly low back pain (LBP) is now the world’s leading cause of morbidity [1]. A tiny proportion of LBP is caused by inflammatory disease or fracture. Most is mechanical LBP whose aetiology is complex and multifactorial with lumbar disc degeneration a significant contributor [2].

Modic change (MC) describes a lesion in the bone marrow of the vertebra adjacent to the endplate. While associated with disc degeneration [3], it is an independent risk factor for LBP [4] and often associated with poor LBP prognosis [2, 5]. MC is indicated by signal change on spine MRIs [6, 7], occurring in 43–81% of LBP patients [2, 8]. There is a genetic component: MC heritability estimates are 30% [9]. Three types of MC have been identified [6, 7]. Type 1 (MC1) is associated with bone marrow and endplate inflammation and oedema; fibrovascular granulation tissue forms and endplate fissure [10, 11]. In type 2 (MC2), healthy, red, haemopoietic bone marrow cells change to yellow, fatty marrow [11]. MC2 endplates show increased reactivity to bone and granulation tissue [10]. Type 3 (MC3), the rarest, indicates bone sclerosis MC is progressive; a cohort of lumbar disc herniation patients showed an increasing prevalence (9–29%) in MC1 over 14 months – whereas MC2 and MC3 did not increase [10].

Occult, or sub-clinical bacterial infection has been proposed to initiate and accelerate disc degeneration pathology. An infective aetiology has been proposed with several reviews linking microorganisms and disc degeneration [12, 13] or MC [2, 11, 14–17] though most investigations of infective disc degeneration are of small sample size and methods vary widely [16–19]. Two meta-analyses have implicated bacteria in disc pathophysiology. The pooled infection rate of nine studies was 36.2% [16] and of 12 studies 25.3% [18]; both analyses found *Cutibacterium acnes* (the bacterium formerly known as *Propionibacterium acnes*) the predominant disc resident.

*C. acnes* is a Gram-positive, facultative, aerotolerant anaerobe, non-spore-forming, rod bacterium [20, 21]. As a commensal, it colonises the skin, oral cavity, gastro-intestinal tract and genitourinary tract; it is, however, an opportunistic pathogen in skin, soft tissue and medical device implantation infections [20, 21]. Selective bacterial culture requires specific (plate or broth) growth media and environments, to which sample cells are added. Moreover, selective culture precludes the opportunity to isolate non-*C*. *acnes* bacteria. DNA based approaches, such as PCR for the 16S rRNA gene can be targeted to identify a single species or can be used more generically, with universal primers to capture a snapshot of all bacterial DNA present in a sample.

### Rationale

This review aims to expand and investigate the occult infection in disc degeneration and MC. We aim to clarify if *C. acnes* is indeed the predominant species as previous work has implied and assess the utility of current laboratory and research practices in the detection and quantification of bacteria in disc material. Viral microorganisms have also been proposed to contribute to disc degeneration [22]; however, detailing viral or fungal pathogenesis is beyond the scope of this review.

### Objectives

Studies of participants who underwent disc excision surgery with subsequent assessment of disc tissue for bacterial growth were included. We investigated whether discs adjacent to MC are at increased risk of bacterial proliferation. Only studies that explicitly stated removal and examination of discs from human participants were included. Cross-sectional and longitudinal observational studies were included.

## Methods

A systematic review protocol was developed in accordance with PRISMA guidelines [23] but was not registered nor is accessible. Four electronic databases were searched: MEDLINE (Ovid), PubMed, Scopus (Ebesco) and Web of Science. The search was conducted on 02.03.21 and corroborated by two authors (IGS and PW). Articles to be included were agreed and a third author (FW) helped finalise decisions lacking consensus. Cited by and reference list searches of included articles were conducted, and a secondary search using Google Scholar was performed. Inclusion criteria specified original research articles published between 2001 and 2021 reporting both human spine disc surgery and the examination of disc bacteria in the context of occult infection. We excluded articles dealing with known infective aetiology (spondylodiscitis, post-operative infections). Exclusion criteria specified abstracts, case reports, editorials, letters, meta-analyses and reviews.

## Results

**Fig. 1:**
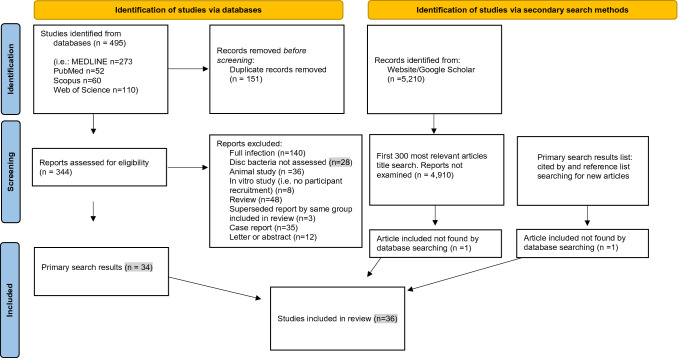
PRISMA flow diagram for evidence for infection in intervertebral disc degeneration: a new systematic review including searches of databases and other sources *Adapted from:* Page MJ, McKenzie JE, Bossuyt PM, Boutron I, Hoffmann TC, Mulrow CD, et al. The PRISMA 2020 statement: an updated guideline for reporting systematic reviews. BMJ 2021;372:n71. https://doi.org/10.1136/bmj.n71

A total of 495 articles were retrieved from four databases, 151 duplicates were removed. Title and abstract searching revealed 155 candidate papers, of these 120 were rejected after full text examination. The cited by search and Google Scholar search each contributed one new article. Figure 1 shows the PRISMA flow diagram and Figure 1 Supplementary (S1) details the search strategy and results. Thirty-six articles were included from 34 research studies; 27 reported finding bacteria in degenerate disc space, nine attributed bacterial findings to contamination. The results are shown in Table 1.

**Table 1:** Summary of studies included

***Studies finding bacteria in disc material* = *28***	***Participants***	***Bacteria identified***	***Lab techniques***	***Control group/control samples***	***Other measures***	***Results***
*(Agarwal, Golish *et al*. 2011)*	*52 single-level lumbar microdiscectomy patients* *No antibiotic use exclusion criteria noted*	*C. acnes* *Peptostreptococcus spp.* *S. aureus* *CoNS spp.*	*Routine bacterial culture Incubated for 5d under standard anaerobic conditions*	*No control or comparison group created & no control specimens taken*	*No host markers of infection assessed. Duration of LBP symptoms and prior surgeries (n* = *11) recorded*	*10/52 (19%) patients had positive cultures: C. acnes (predominantly)*
*(Aghazadeh, Salehpour *et al*. 2017)*	*120 lumbar disc herniation patients (87 MC)* *Antibiotic use 1mth prior to surgery excluded*	*C. acnes* *CoNS* *Gram-negative bacilli Micrococcus* *Corynebacterium* *Neisseria spp.*	*Anaerobic & aerobic culture incubation glovebox each for 7d. Sub-culture and Gram-staining to identify C. acnes* *Specific C. acnes 16S rRNA PCR primers*	*No control or comparison group created. Paravertebral muscle (control) samples taken*	*No host markers of infection assessed. No pain scores assessed*	*60 patients including 42 MC had positive cultures. Predominantly C. acnes was found*
*(Albert, Lambert *et al*. 2013)*	*61 single-level lumbar disc herniation surgery patients* *Antibiotic use 14d prior to surgery excluded*	*C. acnes* *CoNS* *Gram-positive cocci* *Gram-negative rod* *Neisseria species*	*5 tissue samples collected from each patient. Columbia blood agar plates, aerobic & anaerobic incubation for 7d. Presumptive C. acnes* *16S PCR rRNA priming & amplification*	*No control or comparison group created & no control specimens taken; longitudinal study with repeat measures*	*No host markers of infection assessed. FU MRIs conducted*	*28 patients with positive cultures, 80% of anaerobic bacterial positive culture patients developed new MC within* ~ *1.5 years. Bacterial proliferation in disc increased risk of developing new MC*
*(Arndt, Charles *et al*. 2012)*	*83 lumbar disc replacement (degeneration) patients (32 MC1 & 25 MC2)* *No antibiotic use exclusion criteria noted*	*C. acnes* *CoNS* *S. aureus* *Enterobacter cloacae* *Enterobacter aerogenes* *Escherichia coli* *Micrococcus* *Corynebacterium minutissimum* *Corynebacterium coyleae* *Microbacterium* *Brevibacterium* *Rothia dentocariosum* *Enterococcus faecalis* *Streptococcus intermedius*	*Disc samples divided into three parts for analysis, 3 anaerobic media plate cultures 5d with supplementation. Peptone glucose yeast broth for 10d. Plates & broth screened daily for growth*	*No control or comparison group created & no control specimens*	*Additional histological examination showed host inflammatory cells in 33% of positive culture & 5% of negative culture specimens*	*40/83 had positive cultures. Males and MC2 higher rates of microbiological findings. Bacteria in almost half disc & predominantly in males. No correlation with MC1, positive cultures twice as prevalent in MC2 participants*
*(Bivona, Camacho *et al*. 2021)*	*96 anterior cervical discectomy fusion patients* *165 discs* *Long-term antibiotic use excluded*	*C. acnes* *CoNS* *Staphylococcus spp.* *Stretococcus spp.* *Kocuria rhizophila*	*Aerobic & anaerobic 5d culturing. If growth; identified subcultures & Gram-staining, followed by MALDI-TOF MS*	*No control or comparison group created. Logus colli muscle (control) specimens taken*	*FU assessment of surgical success. No host markers of infection assessed. No pain scores assessed*	*Discs with positive control were excluded. 24/83 (29%) bacterial positive. Only study to report Kocuria rhizophila in disc material*
*(Capoor, Ruzicka *et al*. 2016)*	*290 lumbar disc herniation patients* *290 (caudal to avoid statistical bias) discs* *Antibiotic use 1mth prior to surgery excluded*	*C. acnes* *CoNS* *Alpha-haemolytic streptococci*	*Two disc samples, one for culturing undertaken 2 h after acquisition (specimens not frozen). Anaerobic culturing 14d. Frozen sample PCR*	*‘Infective’ (*≥ *1000 CFU/ml) C. acnes group compared with* < *1000 CFU/ml or C. acnes negative. No control specimens*	*Pre-operative clinical data captured (straight leg tests, sensory & motor assessments). No host markers of infection assessed. No pain scores assessed*	*C. acnes identified in 115 (40%) of samples, at an ‘infective’ level in 39 (11%)*
*(Capoor, Ruzicka *et al*. 2017)*	*368 lumbar disc herniation patients* *368 discs* *Antibiotic use 1mth prior to surgery excluded*	*C. acnes* *Staphylococcus saccharolyticus* *Staphylococcus epidermidus* *Staphylococci heamolyticus*	*Extended anaerobic culture. MALDI-TOF. C. acnes genotyping. C. acnes specific 16S probe for FISH & DNA dye for CLSM*	*‘Infective’ (*≥ *1000 CFU/ml) C. acnes group compared with* < *1000 CFU/ml or C. acnes negative. No control specimens*	*FISH/CLSM visualisation of host inflammatory cells & bacterial load assessed. No pain scores assessed*	*162/368 positive for bacterial growth; 119 were C. acnes. No predominance of any C. acnes phylotype. C. acnes seen ‘*in situ*’ within biofilm*
*(Chen, Wang *et al*. 2018)*	*32 cervical fusion patients (21 MC, 28 degenerative disc & 4 trauma patients)* *66 discs* *Antibiotic use 1mth prior to surgery excluded*	*CoNS* *C. acnes* *Staphylococcus epidermidis* *Staphylococci haemolyticus* *Staphylococci capitis*	*Tryptone soy broth and 14 day sealed anaerobic bag incubation. Negative control samples (no tissue) also cultured. Gram-staining and PCR*	*Degenerate disc and trauma control groups. Sternocleidomastoid muscle specimens*	*FU assessment of surgical success. No host markers of infection assessed. No pain scores assessed. Disc herniations classified 1–4 severity, based on MRI*	*9 discs from 8 patients were 16S positive. Infection in degenerate cervical discs associated with younger age, and complete annulus tear but not MC*
*(Coscia, Denys *et al*. 2016)*	*87 patients* *169 discs (30 cervical herniation, 30 lumbar herniation, 30 lumbar discogenic pain, 30 scoliosis control discs & 45 trauma/ deformity control discs)* *No antibiotic use exclusion criteria noted*	*C. acnes* *CoNS*	*Traditional anaerobic culture & Gram-staining*	*5 comparison groups of discs created, importantly 2 non-degenerative control groups. No comparison specimens*	*WBC assessed & histological examination undertaken. No identification of microorganisms, host inflammation or infection with histology. MRI assessment of 27 patients (41 discs). # MC not published*	*Positive cultures found in 45% of discs, sub-clinical infection occurred at a much higher rate in herniation than control patients. No bacterial correlation with MC. Researchers did not separate bacteria from biofilm, perhaps explaining histology findings. Microbes cultured at higher rates in degenerate discs than control specimens*
*(Drago, Romano *et al*. 2020)*	*39 LBP surgery patients (16 MC2 & 23 MC-free)* *No antibiotic use exclusion criteria noted*	*C. acnes* *Bacillus spp.* *Lactobacillus spp.* *Staphylococcus hominis*	*Nucleus pulpous samples. Cultured & incubated 48 h/15d. Vitek 2 microbial identification*	*MC2 compared with MC-free patients. No comparison specimens*	*Full blood count, ESR, CRP & serum electrophoresis*	*6 (37.5%) MC2 samples and 1 (4.3%) MC-free sample cultured positive*
*(Fritzell, Bergström *et al*. 2004)*	*10 lumbar disc herniation patients w. large protrusions/extrusions* *No antibiotic use exclusion criteria noted*	*Bacillus cereus* *Citrobacter braaki* *Citrobacter freundii*	*16S rRNA PCR*	*No control or comparison group created & no control specimens*	*No host markers of infection assessed. Pain assessed pre-operatively & 6 weeks post*	*Small pilot study. 3 specimens from 2 patients were 16S positive*
*(Georgy, Vaida *et al*. 2018)*	*48 cervical surgery patients (13 MC1)* *No antibiotic use exclusion criteria noted*	*C. acnes*	*Aerobic and anaerobic culture plates (4 different agars) incubated for 7d*	*MC1 compared with other degenerative disc cases. No control specimens taken*	*No host markers of infection assessed. No pain scores assessed*	*54% MC1 and 20% MC1-free samples C. acnes positive* *C. acnes in degenerative disc material. MC1 discs affected at a higher rate*
*(Javanshir, Salehpour *et al*. 2017)*	*145 patients (25 cervical & 120 lumbar herniation)* *Antibiotic use 1mth prior to surgery excluded*	*C. acnes*	*Aerobic & anaerobic blood agar glove box, 7d incubation. Sub-culturing to CBA plates, 24 h incubation. Gram-staining all colonies with presumptive C. acnes rapid ID kit. Specific C. acnes 16S PCR primers*	*Compared C. acnes proliferation between cervical and lumbar herniation. No control specimens taken*	*No host markers of infection assessed. No pain scores assessed*	*55 (38%) C. acnes positive. No difference in bacterial positivity between cervical and lumbar disc samples. Sub-clinical disc infection not isolated to lumbar spine*
*(Najafi, Mahmoudi *et al*. 2020)*	*37 lumbar herniation with MC patients* *Antibiotic use 60d prior to surgery excluded*	*C. acnes*	*Culturing and PCR C. acnes specific primers*	*No control or comparison group created & no control specimens*	*VAS & disability scores taken prior to surgery. No host markers of infection assessed*	*23 (62%) bacteria positive, with no difference between disc protrusion, extrusion or budging. No association between VAS or disability scores and bacterial findings*
*(Ohrt-Nissen, Fritz *et al*. 2018)*	*65 (51 lumbar herniation (H) & 14 control (trauma surgery) patients (C))* *Excluded if antibiotic used for 14d within 6mth of surgery*	*(H) C. acnes* *(H&C) Staphylococcus epidermidis* *(H&C) Staphylococcus capitis* *(H) Micrococcus luteus* *(H) Gemmiger formicilis* *(H) Kocuria dechangensis* *(C) Faecalibacterium prausnitzii* *(C) Staphylococcus aureus* *(C) Bacillus simplex*	*16S rRNA PCR and BLAST* *Bacterial aggregates and host inflammatory cells examined with FISH/CLSM*	*Control participants group. No control specimens taken*	*FISH/CLSM analysis, visualised host inflammatory cells. No pain assessments taken*	*16S rRNA detected in 16/51 cases & 7/14 controls. Bacterial aggregates & host inflammatory cells observed in bacterial positive cases only & not in control samples*
*(Rajasekaran, Tangavel *et al*. 2017)*	*22 patients (15 herniation, 5 degenerative & 2 non-degenerative). All disc from lumbar spine* *No antibiotic use exclusion criteria noted*	*73 bacterial proteins identified including 53 C. acnes & 17 S. epidermidis specific proteins*	*Dual 16S rRNA universal primer & proteomic analysis of host defence proteins*	*Non-degenerative control participants. Herniated and degenerated discs compared. No control specimens taken*	*Proteomics evaluation assessed host defence proteins. No pain assessments taken*	*Host defence signature responses to disc herniation and degeneration* *specific bacterial proteins identified in degenerate disc material. Host defence proteins suggestive of infection*
*(Rajasekaran, Soundararajan *et al*. 2020)*	*24 participants (8 MRI healthy, 8 disc degeneration & 8 disc herniation)* *No antibiotic use exclusion criteria noted*	*424 different microbial species. Highly abundant phyla: Proteobacteria, Parcubacteria, Firmicutes, Cyanobacteria & Actinobacteria*	*Genomic DNA extraction and universal amplification (V1-V9 16S rRNA primers)* *Proteins: Mass spectrometry analysis. Proteomics analysis*	*3 groups compared: healthy, degenerated and herniated discs. No control specimens taken*	*Proteomics evaluation assessed host defence proteins. No pain assessments taken*	*Microbiome signatures for healthy, degenerated and herniated discs*
*(Rollason, McDowell *et al*. 2013)*	*64 lumbar disc herniation patients* *Antibiotic use 14d prior to surgery excluded*	*C. acnes* *Predominance of phylotype strains II & III in disc material* *Presumptive C. acnes & Staphylococcus spp. identified* *S. aureus*	*Nucleus extracted from disc sample, disc dissected into five other parts. Aerobic & anaerobic incubation 7d Nucleotide sequencing of recA housekeeping gene to differentiate C. acnes phylotypes multiple disc samples analysed including separate nucleus analysis*	*No control or comparison group created & no control specimens*	*No host markers of infection assessed. No pain scores assessed*	*24 (38%) C. acnes growth* *28% isolates type I A* *9% isolates type I B* *52% isolates type II* *11% isolates type III* *C. acnes phylotypes in disc differ from those on the skin*
*(Salehpour, Aghazadeh *et al*. 2019)*	*120 single-level lumbar disc herniation patients* *Antibiotic use 1mth prior to surgery excluded*	*C. acnes*	*Blood agar plates, 7d aerobic & anaerobic glovebox. Sub-cultured & 24 h anaerobic incubation. Presumptive C. acnes rapid ID followed by 16S rRNA PCR*	*No control or comparison group created & no control specimens*	*No host markers of infection assessed. No pain scores assessed* *Study went on to examined C. acnes resistance to several different antibiotics*	*60 (50%) samples were positive for microorganisms* *study designed to assess C. acnes response to variety of antibiotic drugs*
*(Singh, Siddhlingeswara *et al*. 2020)*	*20 LBP MC patients* *No antibiotic use exclusion criteria noted*	*Identified 16S rRNA gene positive disc specimens*	*16S rRNA Universal eubacteria nested amplification protocol*	*No control or comparison group created & no control specimens*	*Measured or leucocytes, ESR and CRP taken. No pain scores assessed*	*18 (90%) samples demonstrated 16S rRNA gene presence*
*(Stirling, Worthington *et al*. 2001)*	*140 sciatica & LBP patients* *36 discectomy (severe sciatica)* *No antibiotic use exclusion criteria noted*	*C. acnes* *CoNS* *Corynebacterium propinquum*	*Incubated & sub-cultured in broth for 2, 7 & 21d. Gram-staining for microorganisms. Measured C. acnes CFU in positive samples*	*Compared serology inflammatory markers in moderate and extreme sciatica patients. No control specimens taken*	*Serum IgG titres relative to lipid S antigen & CRP levels assessed. Undertook clinical assessment, no pain measures reported*	*19/36 (53%) positive cultures, 16/19 (84%) C. acnes identified in disc samples. First study to link sub-clinical infection with disc pathology. Higher rate of inflammatory serology associated with more severe sciatica and need for surgery*
*(Tang, Wang *et al*. 2018)*	*80 LBP discectomy patients (25 MC)* *Antibiotic use 1mth prior to surgery excluded*	*C. acnes* *CoNS*	*5 disc segments: 3 culture media plates & 2 enriched broth. Results read at 7 & 14d. If bacterial growth; universal primers & 16S rRNA PCR used for identification*	*MC compared with MC-free samples. Surrounding muscle & ligament samples taken*	*Measured severity of disc degeneration. VAS pain measure*	*23 samples positive (3 others excluded, suspicious for contamination). Higher rates of positive cultures in MC samples. No relationship between degeneration severity, nor VAS & bacterial infection*
*(Tang, Chen *et al*. 2019)*	*179 single-level lumbar disc herniation patients* *Antibiotic use 1mth prior to surgery excluded*	*C. acnes* *CoNS*	*3 culture media plates, 2 broth—aerobic & anaerobic culturing for 7 & 14d. Bacterial growth 16S rRNA PCR*	*Participants compared by age & grouped according to severity of disc degeneration. Surrounding muscle & ligament samples taken*	*Intervertebral disc height measured (degeneration severity). No pain measures reported*	*33 samples had positive bacterial growth (6 others excluded, suspicious for contamination). Higher infection rates in younger participants & in those with more degenerated discs*
*(Withanage, Pathirage *et al*. 2019)*	*101 lumbar disc herniation patients* *Antibiotic use 14d prior to surgery excluded*	*CoNS sup* *C.acnes* *Gemella morbilorum* *Staphylococci spp.*	*Enrichment broth & 3 aerobic media cultures followed by additional enrichment and incubation. 3 anaerobic media cultures for 2, 7 & 21d*	*Skin scapings & muscle biopsy control samples taken. No control or comparison group created*	*No host markers of infection assessed. No pain scores assessed*	*18 disc samples positive, 12 for aerobes (CoNS), 6 for anaerobes. First study to identify Gemella morbilorum in disc material. No control samples microbe positive*
*(Yuan, Zhou *et al*. 2017)*	*76 LBP and/or sciatica discectomy patients (70 herniation)* *76 discs* *Antibiotic use 1mth prior to surgery excluded*	*C. acnes* *3 unidentified species*	*Soy broth culture with serum anaerobic glovebox for 14d* *C. acnes specific primer & 16S rRNA PCR*	*Surrounding muscle tissue samples taken. No control or comparison group created*	*WBC counts taken, MRI signs of discitis assessed, other infection signs (fever/chills) assessed. No pain measures reported*	*23/76 samples showed anaerobic growth, 20 C. acnes, 4 of these samples were considered contaminated*
*(Yuan, Chen *et al*. 2018)*	*Sub-set from Yuan, Zhou *et al*. 2017* *15 C. acnes positive* *& 15 C. acnes negative discs*	*NA*	*DNA extracted with boiling and bands visualised with UV photography*	*C. acnes positive samples matched with negative samples for cytokine analysis. Samples from Yuan 2017*	*Histological disc examination & cytokine quantified in disc tissue. Measures of TFN-a, IL-1b, IL-6, IL-8, MCP-1, MIP-1a, IP-10 & neutrophils*	*Visible bacteria present in 7 C. acnes positive and no C. acnes negative specimens. Little correlation between inflammatory markers and C. acnes positivity; only IL-8, MIP-1a & neutrophils significant*
*(Zhou, Chen *et al*. 2015)*	*46 LBP/sciatica patients (MC1 5, MC2 13)* *Antibiotic use 1mth prior to surgery excluded*	*C. acnes*	*Soy broth culture with serum incubated in anaerobic glovebox for 14d followed by* *16S rRNA PCR with C. acnes specific primers*	*Samples with annular tear compared to those without. Surrounding muscle control specimens taken*	*Disc height measured. No host markers of infection assessed. No pain scores assessed*	*11 (23.9%) discs tested positive for 16S rRNA. Only discs with annular tears tested positive. No relationships between MC or sciatica & C. acnes in the disc found*

***Evidence that spinal disc material is sterile n* = *9***	***Participants***	***Bacteria identified***	***Lab techniques***	***Control group/control samples***	***Other measures***	***Results***
*(Ahmed-Yahia, Decousser *et al*. 2019)*	*45 lumbar spine surgery patients (24 MC1, 8MC2)* *77 discs* *Excluded if antibiotic used for 15d within 3 m of surgery*	*C. acnes* *Staphylococcus epidermidis* *C. avidum* *Staphylococcus spp.* *Streptococcus spp.*	*Chocolate culture media for 5 and 10 days followed by broth if clouded. Bacterial identification with MALDI-TOF MS. Universal 16S rRNA PCR (*+ *18S)*	*Compared anterior (58) and posterior (19) approaches for spine surgery*	*Pain duration & VAS recorded. No host markers of infection assessed*	*12/77 (15.6%) specimens were culture positive. Disc bacterial positivity attributed to posterior surgical approach. No difference in MC 0, 1 or 2 culture rates*
*(Alamin, Munoz *et al*. 2017)*	*44 lumbar herniation patients (7 MC1, 4 MC2)* *No antibiotic use exclusion criteria noted*	*None*	*qPCR*	*No control or comparison group created & no control specimens*	*No host markers of infection assessed. No pain scores assessed*	*No evidence of bacterial gene in excised disc samples*
*(Alexanyan, Aganesov *et al*. 2020)*	*64 degenerative lumbar spine disease patients (64 MC)* *80 discs*	*C. acnes*	*Aerobic & anaerobic culture. VITEK 2 microbial identification. Staining & electronic microscopy*	*No control or comparison group created & no control specimens*	*Histological assessment of samples. No pain scores assessed*	*1/64 (1.6%) patient had disc tissue with bacteria identified. No histological confirmation of bacteria or host inflammation*
*(Ben-Galim, Rand *et al*. 2006)*	*30 LBP or sciatica lumbar excision patients* *120 discs* *Antibiotic use 14d prior to surgery excluded*	*CoNS*	*Multiple culture mediums, anaerobic incubation 14d*	*No control or comparison group created & no control specimens*	*No host markers of infection assessed. No pain scores assessed*	*4 disc specimens from 2 participants grew CoNS cultures*
*(Carricajo, Nuti *et al*. 2007)*	*54 lumbar disc herniation patients* *Antibiotic use history recorded – not excluded*	*C. acnes* *Anaerobic streptococci* *Actinomyces sup* *CoNS*	*4 culture media 10d/20d*	*Surrounding tissue & environment control taken. No control or comparison group created*	*CRP & WBC levels evaluated. No pain scores assessed*	*Positive cultures in 2 (3.7%) disc samples and 10 (18.5%) muscle controls. Surgery air samples also positive for C. acnes*
*(Fritzell, Welinder-Olsson *et al*. 2019)*	*60 participants (40 LBP/ herniation & 20 scoliosis control) (MC 23/40, 18/20)*	*C. acnes* *Streptococcus spp.* *Lactococus lactis* *Corynebacterium* *Burkholderiales*	*Culturing & universal 16S rRNA PCR*	*Non-degenerative disc controls included. Skin & surrounding tissue samples taken*	*No host markers of infection assessed. No pain scores assessed*	*2 participants with disc only positive samples. 85% participants had one or more samples positive for bacteria. High rates in muscle & skin control samples. No difference between herniation and scoliosis groups in positive disc samples. No association between MC and positive bacterial growth*
*(Li, Dong *et al*. 2016)*	*22 lumbar disc herniation patients (2 MC1, 6 MC2)* *30 discs* *Antibiotic use 1mth prior to surgery excluded*	*CoNS* *Staphylococcus epidermidis*	*Only used nucleus pulpous. Aerobic & anaerobic culturing 10d incubation. Identified with Gram-staining, morphology, oxygen tolerance & Api20A*	*Paravertebral muscle control samples taken. Comparison groups of degeneration according to Pfirrmann classification*	*No host markers of infection assessed. No pain scores assessed*	*3/30 specimens positive for bacteria. No association with MC*
*(Rao, Maharaj *et al*. 2020)*	*812 participants (550 disc & paraspinal samples, 191 disc only, 46 sham; paraspinal only & 25 control; trauma patients)* *No antibiotic use exclusion criteria noted*	*Acinetobacter* *Candida* *Corybacterium* *Cutibacterium (C. acnes) Staphylococcus*	*Aerobic & anaerobic culture: 4 different plates for 7 & 14 days*	*Non-degenerate controls used. Surrounding fat, ligament and muscle tissue control samples taken*	*Sub-section of patients’ samples underwent histological examination for inflammation evidence. No pain scores assessed*	*Positive cultures in discs: 33/191 (17%) ‘disc only’, 146/554 (27%) ‘disc* + *control’, 12/25 (48%) control group samples. High rates of positive bacteria in paravertebral control samples. Largest study investigating bacteria in disc pathology, findings suggest contamination*
*(Rigal, Thelen *et al*. 2016)*	*313 participants (303 MC1, 58 MC2)* *385 discs* *No antibiotic use exclusion criteria noted*	*C. acnes* *S. epidermidis* *Citrobacter freundii* *Saccharopolyspora hirsuta*	*Anaerobic culture 15d. Plates/growth then underwent histological examination*	*No control specimens taken*	*Life & health satisfaction scales, pain rating scales at BL, 3,6 & 12 mths. Histological examination of disc tissue for host inflammatory response & neutrophils*	*6/385 (1.5%) samples positive cultures. The origin of inflammation in disc degeneration is unknown. No association between bacteria positive disc and outcome*

### Studies finding bacteria in disc material

Stirling’s work first identified raised IgG antibodies in 43/140 (31%) patients with sciatica or LBP. Severely affected patients underwent microdiscectomy, and bacteria was detected in 19/36 (53%) samples [24]. *C. acnes* 16/19 (83%), coagulase-negative staphylococci (CoNS) 2/19 (11%) and *Corynebacterium propinquum* 1/19 (5%) were cultured [24]. A high proportion of positive serology tests were from patients with microorganism positive disc samples [24]. Fritzell and colleagues found 2/10 (20%) degenerate disc samples tested positive for *Bacillus cereus*, *Citrobacter braakii* and *C. freundii*, and asserted these were ‘true findings’ based on their strict collection methodology [25]. Similarly, *C. acnes* (7/52, 13.5%), *Peptostreptococcus* spp. (1/52, 2%), *Staphylococcus aureus* (1/52, 2%) and CoNS (1/52, 2%) were cultured from lumbar herniation microdiscectomy specimens [26]. Yuan and colleagues (2017) found *C. acnes* with culture and selective PCR in 16/76 (21%) discs, having discarded four positive disc samples with a corresponding positive muscle control sample [27]. Histological examination confirmed rod-shaped bacteria in half the PCR positive samples and no randomly selected PCR negative samples [27].

Contamination may be distinguished from infection by bacterial quantification—counting colony forming units (CFU). Proposing ≥ 1000 CFU/ml indicates active infection, *C. acnes* (115/290), CoNS (31/290) and alpha-haemolytic streptococci were isolated after disc herniation material homogenisation and qPCR amplification [28]. Bacteria were identified in 45% of samples; 11% positive for *C. acnes* ≥ 1000 CFU/ml [28]. Adding to the cohort and improving methodology by incorporating mass spectrometry, a second study found *C. acnes* ≥ 1000 CFU/ml in 10% of 368 patients’ samples [29]. *Staphylococcus*, *Streptococcus* and *Corynebacterium* ≥ 1000 CFU/ml were detected in 13 samples [29]. Another analysis of herniated disc samples, with a lower ‘infective’ CFU criteria reported *C. acnes* 24/64 (38%), CoNS 5/65 (8%) and Gram-negative diplococci 1/64 colonisation of 1–150 CFU per sample [30]. Another investigation reported a predominance of CoNS in 7/66 (11%) and *C. acnes* in 2/66 (3%) cervical disc samples using anaerobic culturing and PCR [31]. Withanage and colleagues (2019) cultured CoNS, *C. acnes* and *Gemella morbillorum* in 18/101 (18%) lumbar herniation samples [32].

Salehpour and co-workers found bacteria in 60/120 (50%) lumbar herniation discs; positive samples were screened for *C. acnes* with a kit and PCR. *C. acnes* accounted for 77% cases of bacterial growth and researchers went on to investigate *C. acnes* sensitivity to antibiotics [33]. Recently, 96 cervical degeneration patients were recruited and 55% of all disc samples grew positive cultures [34]. Disc samples with a corresponding bacterial positive muscle biopsy were eliminated from analysis, leaving 17/96 (18%) of participants with a positive disc sample and a ‘clean’ control sample. Predominantly *C. acnes* along with CoNS, *Staphylococcus* spp*., Streptococcus* spp*.* and one example of *Kocuria rhizophila* was cultured in disc specimens [34].

#### Modic change

Eight articles found higher rates of positive bacterial samples came from MC patients than participants without MC. For example, 54% of patients with MC1 were positive for the presence of *C. acnes* compared to 20% of patients without MC1, in a study of 48 cervical biopsy samples [35]. Four studies combined samples from MC1 and MC2 patients; Aghazadeh and colleagues (2017) reported 80% of MC samples were *C. acnes* positive while only 14% of MC-free samples were positive [36]. Yuan and co-workers (2018) found 12/15 (80%) of *C. acnes* positive cultures were from MC participants; however, the small sample size of this study made drawing statistically based conclusions difficult [37]. Tang and colleagues (2018) reported 26/80 (33%) herniation samples were positive for bacteria using PCR. One positive sample excluded from subsequent analysis which showed 15/25 (60%) patients with disc bacteria had MC [38].

Disc bacteria was recorded in recent studies of MC patients (type unspecified) [39, 40]. Singh and co-workers (2020) reported 90% of 20 LBP; MC participants’ samples were positive for 16S rRNA gene with PCR. This investigation found evidence of inflammation associated with MC; participants had raised levels of leucocytes, ESR and CRP [39]. Najafi and colleagues (2020) found a high rate of *C. acnes* prevalence via culturing in 23/37 (62%) samples from LBP patients [40].

Two studies reported occult infection in MC2 adjacent discs. Arndt and co-workers (2012) did not find differences when MC1 and MC2 were pooled and compared to MC-free samples; however, MC2 specimens alone showed greater bacterial positivity than other samples [41]. Drago and colleagues (2020) concluded MC2 may be associated with occult infection when samples from 6/16 (37.5%) MC2 patients and only 1/23 (4%) MC-free control returned positive bacterial cultures [42].

One longitudinal study has demonstrated bacterial proliferation precedes MC1 development. In 28/61 (46%), herniation surgery patients positive for microorganisms, 80% developed new MC1 in the subsequent 12–24 months [43]. 44% of negative culture patients developed MC1 giving an OR of 5.6 (95% CI 1.51–21.95) for new MC1 given a positive anaerobic microbe culture [43]. Four articles did not find bacterial abundance differed between MC and MC-free degenerate discs [31, 44–46].

#### Disc herniation and infection

The most included studies used homogeneous groups of disc herniation participants (i.e. all with herniated discs). Four studies compared and reported increased abundance and/or species differences in herniated over non-herniated discs [44, 46–48]. Coscia and co-workers (2016) found positive bacterial cultures in 76/145 (45%) of excised disc material, but notably, 32/61 (52%) herniation patients versus 19/77 (25%) controls samples were positive for *C. acnes* (45%), CoNS (40%) and various species (4%) [46]. Two studies included patients with herniated and non-herniated discs but did not report and bacterial abundance differences [25, 37].

Two studies reported a positive relationship between herniation severity and *C. acnes* proliferation. Zhou and colleagues (2015) found 10/28 (36%) discs supported *C. acnes* proliferation when the protective annular was torn [45]. Only 1/18 (6%) untorn disc did, however, this specimen was removed from the analysis as its corresponding muscle control sample was also culture positive [45]. Chen and co-workers (2018) found more positive cultures in more damaged discs that were extruded or sequestered than those budging or protruded [31]. Yet Najafi and colleagues (2020) found no bacterial differences between disc pathologies of extrusion, protrusion or bulging [40].

#### Patient subgroup infection

Eight studies identified patient subgroups other than those with MC or herniation as susceptible to occult infection. Two studies reported greater bacterial growth in men than women [28, 41] while one reported higher rates of infection in disc samples from women [34]. Two research groups reported higher rates of disc infection in younger participants [28, 29, 38, 49], Yet Najafi and co-workers (2020) found specimens from older patients with MC were more likely to be *C. acnes* positive [40]. Two studies reported increased proliferation of microorganisms differed at spinal levels. Greater *C. acnes* abundance at L4–L5 than other lumbar discs was reported in herniation patients [41] and greater *C. acnes* colonisation in C6–C7 and L4–L5 than other spinal levels was also found [50]. Most articles did not report bacterial differences in sex, age or spinal level subgroups.

#### Disc colonisation by multiple microbes

Of 27 positive studies, five reported finding several species depicting a bacterial ecosystem. Utilising a pre-operative biopsy approach to decease contamination, 40/83 (48%) lumbar disc samples returned positive cultures for *C. acnes* (35%), CoNS (31%), *Staphylococcus aureus* (6%), three species of Gram-negative bacilli (6%), *Micrococcus* spp. (6%), *Corynebacterium* spp. (6%) and single examples of five other species using only routine culturing [41]. Another study found *C. acnes* (38%), CoNS (6%), Gram-negative bacilli (2.5%) *Micrococcus* spp. (4%), *Corynebacterium* spp. (3.5%) and *Neisseria* spp. (2.5%) in 60/120 (50%) herniation samples using Gram-staining culturing and 16S rRNA gene PCR [36]. Ohrt-Nissen and colleagues (2018) listed BLAST scores following genome sequencing and recorded eight species in herniation and six in non-degenerative samples [44]. Using microscopy, they saw host inflammatory cell activation only in degenerate disc samples, not in control samples [44].

Using the agnostic techniques of proteomics and 16S rRNA gene analysis, Rajasekaran’s study of 22 herniation samples demonstrated many more bacterial proteins than either degenerate or discs from participants with a healthy spine [47]. A total of 2061 bacterial proteins were identified, only 178 of these shared between the three disc groups [47]. In a subsequent study using culture-independent 16S rRNA gene sequencing [48] 424 species were reported, including five dominant taxa with abundance ranges from 13 to 16% [48]. Microbiota signatures for both disc degeneration and herniation were characterized as shifts in composition and diversity away from the disc microbiome of a healthy spine [48]. *C. acnes* was found in similar proportions in all disc groups and was not the most dominant bacteria with relative abundance ranging from 1.5 to 3% [48].

### Evidence of contamination

Nine reports attributing bacterial colonisation in disc samples to contamination were found. Ben-Galim and colleagues (2006) cultured multiple samples from 30 LBP patients and found two patients returned four positive CoNS specimens: 4/120 (4%) [51]. Another study involving lumbar herniation patients cultured *C. acnes* in 2/54 (4%) discs, in 10/54 (19%) muscle tissue control and in negative air control samples [52]. Li and colleagues (2016) recruited 22 herniation patients contributing 30 discs specimens, CoNS and *Staphylococcus epidermidis* were cultured from three and one samples, respectively [53]. Another investigation of 44 herniation specimens analysed with a high-quality next generation PCR assay did not detect evidence of the bacterial 16S rRNA gene in samples [54].

*C. acnes* was detected in 7/45 (16%) disc specimens with culturing and PCR, however, 24% of posterior surgical approach samples were associated with positive culture, whereas only 9% of anterior approach samples were [55]. In this study, there were no disc bacterial differences between MC and MC-free patients [55]. Fritzell and colleagues (2019) later study also found no relationship between disc bacterial findings and MC in 2/40 (5%) LBP patients. Bacterial cultures from muscle tissues samples from LBP and control groups returned 30% and 20%, respectively [56]. Using a video assisted minimal skin contact anterior surgical approach, Rigal and co-workers (2016) found 6/313 (2%) disc biopsies from MC patients grew positive bacterial cultures [57]. There was no relationship between bacterial findings and outcomes a year following surgery [57]. The largest disc infection investigation to date collected samples from 812 participants. Contamination (muscle) control samples along with disc samples were taken from disc degenerative (case) and non-degenerative (control) participants [58]. Significantly higher rates of bacteria were cultured from control group samples of muscle and disc (48%) than degenerate disc samples (17%) [58]. Prior surgeries and multilevel surgeries were associated with higher rates of bacterial growth [58]. There was no difference in disc bacterial prevalence between case and control groups, MC or vertebral level treated [58]. Recently, 1/64 MC samples verified *C. acnes* growth from disc material, no patients had any signs of infection [59]. Researchers concluded that infection and *C. acnes* disc penetration must be rare if extant [59].

## Discussion

Articles in this review link occult infection and disc degeneration, finding evidence of bacteria, mainly *C. acnes*, using selective microbial culture and targeted PCR. Targeted culturing predominated with only six studies using universal PCR or genome-wide sequencing techniques [38, 39, 47, 48, 55, 56]. A causal role for bacteria in disc degeneration or MC is not clear, and all but two studies were cross sectional. Causality was suggested by Albert and colleagues (2013) in their longitudinal study which showed discs with bacteria increased the likelihood to develop MC1 in adjacent vertebrae [43], though Rigal and co-workers (2016) saw no influence of their few positive bacterial cases on outcome [57].

MC1 adjacent discs have been proffered as inflammatory and most vulnerable to occult infection [14]; however, only study showed MC1 was associated with increased rates of disc bacterial growth [35]. MC specimens have been reported more infective than MC-free specimens [36–39] as have MC2 samples [41, 42]. Increased immune activation in MC2 compared with MC-free samples has also been found, however, without examination of disc bacteria [60]. MC type is not static, MC1 can progress to MC2, or recover and a significant percentage of MC radiography report mixed types, most commonly a mix of MC1 and MC2 [61]. Thus, MC types are semi-fluid, we therefore cannot dismiss the possibility that MC2 also represents vulnerability to infection, inflammation and advanced disc degeneration.

A mechanistic explanation for occult infection has been proposed: harbouring *C. acnes* in disc tissue corresponded with annular tears, with intact discs not returning positive PCR tests [45].

Chen and colleagues (2018) found more positive cultures in more damaged discs that were extruded or sequestered than those budging or protruded [31]. It is interesting that Ahmed-Yahia and colleagues (2019) concluded high rates of contamination due to surgical approach [55] Samples from participants were assigned to either Group 1 ALIF or disc prothesis (herniation not specified) or Group 2 TLIF for herniated discs. The second group’s higher rate of bacteria positive samples drew the conclusion TLIF was a more contaminating approach—when there may have been disc pathology differences between the two groups.

It is possible that *C. acnes* accesses the nucleus pulpous via disruption to the annulus fibrosus. In rats, MC1-like changes, increased 16S rRNA gene expression and immune activation occurred with injection of *C. acnes* isolates to the disc [62]. In vitro*,* MC adjacent disc cells had an increased inflammatory response to *C. acnes* compared with healthy disc cells [63]. If these findings mirror interactions in the human spine, damaged discs and bacteria may culminate in a synergistic degenerative sequela, producing local inflammation and therefore more pain. Of course, *C. acnes* may not be the only bacterial species that causes inflammation and degeneration when artificially introduced to a disc.

While this review found evidence to support occult infection in degenerate discs, particularly *C. acnes*—it remains unclear the extent to which bacteria reside in the disc space. Wedderkopp and colleages (2009) sampled MC lesions and found only two samples cultured positive for bacteria; however, these two patients were treated with antibiotics which brought one temporarily pain relief [64]. This finding does hint at bacterial involvement in degeneration and pain; however, other research efforts administering antibiotics for back pain have met with varying successes. No antibiotic studies have assessed disc bacterial prevalence, and hence are excluded from this review [65–67]. A recent, interesting review by Gilligan and colleagues (2021) summarises eight antibiotic trials for LBP [68]. These authors conclude a LBP sub-type (likely patients with MC1, wishing to avoid surgery) may be amenable to antibiotic therapy [68]. To enter the bone or disc space antibiotic regimes are necessarily protracted and responsible antibiotic stewardship calls for such treatments to be approached with prudence.

This review highlights the heterogeneity of microbial discovery, identification and sequencing techniques that have biased many reports in this field. Qualifying and quantifying bacteria uniquely associated with disc degeneration and MC is currently incomplete and a primary step in understanding if antibiotic treatment is suitable for LBP. As a handful of studies indicate; bacteria may reside in the healthy disc space [44, 47, 48], an additional reason to proceed with caution. Agnostic examination of disc samples as standard practice will give a better understanding of the commensal and/or pathogenic nature of degenerate disc bacteria. This is needed before antibiotics can reasonably be considered for back pain treatment. While selective and acute bias to identify bacteria in disc specimens persists, such advances are impossible. Bias in this field is underscored by several studies finding a high prevalence of bacteria yet low numbers of species [27, 35, 38, 45, 46, 50]. Immediately storing samples in culture medium [40] for example, selects against most other bacteria, making accurate determination of the whole range of species impossible.

Rao and colleagues (2020) scrupulously collected samples from (control) participants with non-degenerate discs, as well as multiple control specimens from surrounding disc anatomy. Yet no more advanced bacterial identification than microbial culture was undertaken in samples from 812 participants, the largest study of its kind [58]. *C. acnes* specific culturing may explain why discs removed from patients with previous and more extensive surgery had higher rates of positive bacterial culture [58]. Moderately high rates of positive bacterial samples were obtained from discs (17%), (comparable with other studies reporting disc bacteria; see Table 1.), yet almost half muscle control specimens were positive for bacteria [58]. No bacterial differences were found between case degenerate and control discs [58]. In contrast, Coscia and co-workers (2016) showed lower bacterial abundance in non-degenerative disc samples; perhaps curiously, also only using culture identification [46].

Alamin and colleagues (2017) reported a sterile disc environment, despite using a high-quality next generation PCR assay [54]. These results could be due to insufficient DNA extraction from disc samples, a fair possibility as their validation cohort amplicon sequencing worked on cultured samples and found similar pathogenic bacteria from disc material as previous reports [54].

A small number of studies reported an ecosystem of bacteria in degenerate discs [36, 41, 44, 47, 48]. Ohrt-Nissen and co-workers (2018) found a similar diversity of species in healthy and degenerate disc samples. Only finding host inflammatory cell activation in degenerate disc material adds credence to the notion that disc degeneration results from bacterial imbalance rather than from the presence of bacteria themselves [44]. Perhaps not surprisingly, more exploratory technologies found greater diversity of bacterial species. Rajasekaran’s group used proteomics to identify 73 bacterial specific proteins along with upregulation of defence proteins, a host immune counter to bacterial infection [47]. Bacterial proteins unique to degenerate, herniated and healthy discs were found as well as overlaps between disc groups [47]. These data led them to conclude a presence of commensal, albeit low level, human disc bacteria. Support for this idea gained further traction with a second project which used a metagenomic approach to find 424 different species, support for the existence of disc microbiota and a disc microbiome [48]. Higher alpha-diversity and differences in beta-diversity were found in healthy discs compared with degenerated or herniated samples [48]. This paper begs replication—and if this is forthcoming may provide a guide towards future possible LBP treatments.

If bacteria do sequester into the disc space another challenge posed to successful identification is the tendency of bacteria to cluster and grow unevenly distributed within a protective biofilm [14]. Viewing and quantifying bacteria within biofilm may accurately assess colonisation. Two studies used fluorescence in situ hybridization (FISH) staining and viewed samples with specialised confocal laser scanning microscopy (CLSM) [29, 44]. These technologies enable quantifying bacterial biofilm sequestration and growth along with producing high-resolution, three-dimensional pictures of a bacterium in situ [12]. Use of FISH/CLSM permits tissue assessment of uncontaminated samples. Well-established biofilm formation is impossible from the brief skin contact that may occur during spine surgery. FISH/CLSM could offer an important contribution to verifying bacterial colonisation within degenerate disc material.

## Limitations

It is clear why controversy continues to surround the question of occult infection and bacteria in the degenerate disc space. Most protocols kept samples free of contamination, although removing a totally ‘sterile’ disc is difficult if not impossible, thus contamination poses an ongoing challenge. Acquiring healthy human disc specimens is difficult; thus, a rare control group in these investigations. Only two longitudinal examinations were relevant [43, 57], yet knowing what becomes of patients with high bacterial loads in degenerate discs will help determine the pathogenic (or not) nature of such microbes. Only 12 studies used surrounding tissue as negative control samples, routine collection of these samples will help future research clarify the extent of sample contamination. FISH/CLSM is a time-consuming procedure, covering only a fraction of a tissue biopsy [44], specialised equipment and skills are required and only ~ 15% of researchers use it [12]. Nonetheless, proponents have made multiple calls to include FISH/CLSM as standard for disc material analysis [12, 14, 29, 44].

## Conclusion

Evidence in this systematic review implicates *C. acnes* as a bacterial resident in degenerate disc tissue, identified through culture, PCR and microscopy. Some evidence suggests a broad diversity of microbes within the disc. Most laboratory techniques were biased towards identifying *C. acnes*. The field will benefit from new genomic methods which identify bacteria by their genetic material and may be known as well as unknown (i.e. not yet catalogued). The inclusion of omics analyses and advanced histological techniques are not widely used yet to determine sub-clinical infection within the degenerate disc will strengthen such research.

## Key Messages

- Contamination is not an adequate explanation for positive bacterial findings in degenerate disc material.
- Culturing bias towards finding *C. acnes* has overwhelmed research examining occult infection in degenerate discs.
- Agnostic, exploratory disc bacteria assessment may best inform any occult infection and disc degeneration links.
- Insufficient evidence exists to suggest changes to current clinical treatment.

## Supplementary Information

Below is the link to the electronic supplementary material.

Supplementary file1 (DOCX 17 KB)

## Data Availability

Supplementary document provided detailing systematic search strategy.

## Abbreviations

BLAST: Basic local alignment search tool
C6-C7: Disc between cervical vertebrae 6 and 7
CFU: Colony forming unit
CLSM: Confocal laser scanning microscopy
CoNS: Coagulase-negative staphylococci
CRP: C-reactive protein
DNA: Deoxyribonucleic acid
ESR: Erythrocyte sedimentation rate
FISH: Fluorescence in situ hybridization
IgG: Immunoglobulin
L4–L5: Disc between lumbar vertebrae 4 and 5
LBP: Low back pain
MC: Modic change
MC1: Modic change type 1 etc.
MRI: Magnetic resonance image
OR: Odds ratio
PCR: Polymerase chain reaction
rRNA: Ribosomal ribonucleic acid

## References

[1] Hartvigsen J, Hancock MJ (2018). What low back pain is and why we need to pay attention. Lancet.

[2] Dudli S, Fields AJ, Samartzis D, Karppinen J, Lotz JC (2016). Pathobiology of Modic changes. Eur Spine J.

[3] Kjaer P, Korsholm L, Bendix T, Sorensen JS, Leboeuf-Yde C (2006). Modic changes and their associations with clinical findings. Eur Spine J.

[4] Määttä JH, Wadge S, MacGregor A, Karppinen J, Williams FM (2015). ISSLS prize winner: vertebral endplate (Modic) change is an independent risk factor for episodes of severe and disabling low back pain. Spine.

[5] Maatta JH, Karppinen J, Paananen M, Bow C, Luk KDK, Cheung KMC, Samartzis D (2016). Refined phenotyping of Modic changes: imaging biomarkers of prolonged severe low back pain and disability. Medicine (Baltimore).

[6] Modic MT, Masaryk TJ, Ross JS, Carter JR (1988). Imaging of degenerative disk disease. Radiology.

[7] Modic MT, Steinberg PM, Ross JS, Masaryk TJ, Carter JR (1988). Degenerative disk disease: assessment of changes in vertebral body marrow with MR imaging. Radiology.

[8] Arana E, Kovacs FM (2011). Modic changes and associated features in Southern European chronic low back pain patients. Spine J.

[9] Maatta JH, Kraatari M, Wolber L, Niinimaki J, Wadge S, Karppinen J, Williams FM (2014). Vertebral endplate change as a feature of intervertebral disc degeneration: a heritability study. Eur Spine J.

[10] Albert HB, Manniche C (2007). Modic changes following lumbar disc herniation. Eur Spine J.

[11] Georgy M, Stern M, Murphy K (2017). What is the role of the bacterium *Propionibacterium acnes* in type 1 Modic changes? A review of the literature. Can Assoc Radiol J.

[12] Capoor MN, Birkenmaier C (2019). A review of microscopy-based evidence for the association of *Propionibacterium acnes* biofilms in degenerative disc disease and other diseased human tissue. Eur Spine J.

[13] Chen Z, Cao P, Zhou Z, Yuan Y, Jiao Y, Zheng Y (2016). Overview: the role of *Propionibacterium acnes* in nonpyogenic intervertebral discs. Int Orthop.

[14] Manniche C, O'Neill S (2019). New insights link low-virulent disc infections to the etiology of severe disc degeneration and Modic changes. Future Sci OA.

[15] Viswanathan VK, Shetty AP, Rajasekaran S (2020). Modic changes–an evidence-based, narrative review on its patho-physiology, clinical significance and role in chronic low back pain. J Clin Orthop Trauma.

[16] Berjano P, Villafane JH (2019). Is *Propionibacterium acnes* related to disc degeneration in adults? A systematic review. J Neurosurg Sci.

[17] Urquhart DM, Zheng Y (2015). Could low grade bacterial infection contribute to low back pain?. A Syst Rev BMC Med.

[18] Jiao Y, Lin Y, Zheng Y, Yuan Y, Chen Z, Cao P (2019). The bacteria-positive proportion in the disc tissue samples from surgery: a systematic review and meta-analysis. Eur Spine J.

[19] Ganko R, Rao PJ, Phan K, Mobbs RJ (2015). Can bacterial infection by low virulent organisms be a plausible cause for symptomatic disc degeneration? A systematic review. Spine.

[20] Achermann Y, Goldstein EJ, Coenye T, Shirtliff ME (2014). *Propionibacterium acnes*: from commensal to opportunistic biofilm-associated implant pathogen. Clin Microbiol Rev.

[21] Aubin GG, Portillo ME, Trampuz A, Corvec S (2014). *Propionibacterium acnes*, an emerging pathogen: from acne to implant-infections, from phylotype to resistance. Med Mal Infect.

[22] Alpantaki K, Katonis P, Hadjipavlou A, Spandidos D, Sourvinos G (2011). Herpes virus infection can cause intervertebral disc degeneration: a causal relationship? The Journal of bone and joint surgery. Br Volume.

[23] Page MJ, McKenzie JE (2021). The PRISMA 2020 statement: an updated guideline for reporting systematic reviews. BMJ.

[24] Stirling A, Worthington T, Rafiq M, Lambert PA, Elliott TSJ (2001). Association between sciatica and *Propionibacterium acnes*. Lancet.

[25] Fritzell P, Bergstrom T, Welinder-Olsson C (2004). Detection of bacterial DNA in painful degenerated spinal discs in patients without signs of clinical infection. Eur Spine J.

[26] Agarwal V, Golish SR, Alamin TF (2011). Bacteriologic culture of excised intervertebral disc from immunocompetent patients undergoing single level primary lumbar microdiscectomy. J Spinal Disord Tech.

[27] Yuan Y, Zhou Z (2017). Histological identification of *Propionibacterium acnes* in nonpyogenic degenerated intervertebral discs. Biomed Res Int.

[28] Capoor MN, Ruzicka F (2016). Prevalence of *Propionibacterium acnes* in intervertebral discs of patients undergoing lumbar microdiscectomy: a prospective cross-sectional study. PLoS One.

[29] Capoor MN, Ruzicka F (2017). *Propionibacterium acnes* biofilm is present in intervertebral discs of patients undergoing microdiscectomy. PLoS One.

[30] Rollason J, McDowell A (2013). Genotypic and antimicrobial characterisation of *Propionibacterium acnes* isolates from surgically excised lumbar disc herniations. Biomed Res Int.

[31] Chen Y, Wang X (2018). Low virulence bacterial infections in cervical intervertebral discs: a prospective case series. Eur Spine J.

[32] Withanage N, Pathirage S, Perera S, Peiris H, Athiththan L (2019). Identification of microbes in patients with lumbar disc herniation. J Biosci.

[33] Salehpour F, Aghazadeh J, Mirzaei F, Ziaeii E, Alavi SAN (2019). *Propionibacterium acnes* infection in disc material and different antibiotic susceptibility in patients with lumbar disc herniation. Int J Spine Surg.

[34] Bivona LJ, Camacho JE (2021). The prevalence of bacterial infection in patients undergoing elective ACDF for degenerative cervical spine conditions: a prospective cohort study with contaminant control. Global Spine J.

[35] Georgy MM, Vaida F, Stern M, Murphy K (2018). Association between type 1 Modic changes and *Propionibacterium acnes* infection in the cervical spine: an observational study. AJNR Am J Neuroradiol.

[36] Aghazadeh J, Salehpour F (2017). Modic changes in the adjacent vertebrae due to disc material infection with *Propionibacterium acnes* in patients with lumbar disc herniation. Eur Spine J.

[37] Yuan Y, Chen Y (2018). Association between chronic inflammation and latent infection of *Propionibacterium acnes* in non-pyogenic degenerated intervertebral discs: a pilot study. Eur Spine J.

[38] Tang G, Wang Z, Chen J, Zhang Z, Qian H, Chen Y (2018). Latent infection of low-virulence anaerobic bacteria in degenerated lumbar intervertebral discs. BMC Musculoskelet Disord.

[39] Singh S, Siddhlingeswara GI, Rai A, Iyer RD, Sharma D, Surana R (2020). Correlation between Modic changes and bacterial infection: a causative study. Int J Spine Surg.

[40] Najafi S, Mahmoudi P, Bassampour SA, Shekarchi B, Soleimani M, Mohammadimehr M (2020). Molecular detection of Propionibacterium acnes in biopsy samples of intervertebral disc with Modic changes in patients undergoing herniated disc surgery. Iran J Microbiol.

[41] Arndt J, Charles YP, Koebel C, Bogorin I, Steib JP (2012). Bacteriology of degenerated lumbar intervertebral disks. J Spinal Disord Tech.

[42] Drago L, Romano CL, Cecchinato R, Villafane JH, De Vecchi E, Lamartina C, Berjano P (2020). Are Modic type 2 disc changes associated with low-grade infections? A pilot study. J Neurosurg Sci.

[43] Albert HB, Lambert P (2013). Does nuclear tissue infected with bacteria following disc herniations lead to Modic changes in the adjacent vertebrae?. Eur Spine J.

[44] Ohrt-Nissen S, Fritz BG, Walbom J, Kragh KN, Bjarnsholt T, Dahl B, Manniche C (2018). Bacterial biofilms: a possible mechanism for chronic infection in patients with lumbar disc herniation–a prospective proof-of-concept study using fluorescence in situ hybridization. APMIS.

[45] Zhou Z, Chen Z (2015). Relationship between annular tear and presence of *Propionibacterium acnes* in lumbar intervertebral disc. Eur Spine J.

[46] Coscia MF, Denys GA, Wack MF (2016). *Propionibacterium acnes*, coagulase-negative staphylococcus, and the “biofilm-like” intervertebral disc. Spine.

[47] Rajasekaran S, Tangavel C (2017). ISSLS PRIZE IN CLINICAL SCIENCE 2017: Is infection the possible initiator of disc disease? An insight from proteomic analysis. Eur Spine J.

[48] Rajasekaran S, Soundararajan DCR (2020). Human intervertebral discs harbour a unique microbiome and dysbiosis determines health and disease. Eur Spine J.

[49] Tang G, Chen Y, Chen J, Wang Z, Jiang W (2019). Higher proportion of low-virulence anaerobic bacterial infection in young patients with intervertebral disc herniation. Exp Ther Med.

[50] Javanshir N, Salehpour F, Aghazadeh J, Mirzaei F, Naseri Alavi SA (2017). The distribution of infection with *Propionibacterium acnes* is equal in patients with cervical and lumbar disc herniation. Eur Spine J.

[51] Ben-Galim P, Rand N (2006). Association between sciatica and microbial infection: true infection or culture contamination?. Spine.

[52] Carricajo A, Nuti C (2007). *Propionibacterium acnes* contamination in lumbar disc surgery. J Hosp Infect.

[53] Li B, Dong Z (2016). Association between lumbar disc degeneration and *Propionibacterium acnes* infection: clinical research and preliminary exploration of animal experiment. Spine.

[54] Alamin TF, Munoz M (2017). Ribosomal PCR assay of excised intervertebral discs from patients undergoing single-level primary lumbar microdiscectomy. Eur Spine J.

[55] Ahmed-Yahia S, Decousser JW (2019). Is the discopathy associated with Modic changes an infectious process? Results from a prospective monocenter study. PLoS One.

[56] Fritzell P, Welinder-Olsson C (2019). Bacteria: back pain, leg pain and Modic sign-a surgical multicentre comparative study. Eur Spine J.

[57] Rigal J, Thelen T, Byrne F, Cogniet A, Boissiere L, Aunoble S, Le Huec JC (2016). Prospective study using anterior approach did not show association between Modic 1 changes and low grade infection in lumbar spine. Eur Spine J.

[58] Rao PJ, Maharaj M (2020). Degenerate-disc infection study with contaminant control (DISC): a multicenter prospective case-control trial. Spine J.

[59] Alexanyan MM, Aganesov AG, Pogosyan EL, Mrugova TM, Ivanova AG, Chukina MA, Gemdzhian EG (2020). The role of infectious pathogens in etiopathogenesis of degenerative intervertebral disc disease. Hir pozvonoč.

[60] Schroeder GD, Markova DZ (2017). Are Modic changes associated with intervertebral disc cytokine profiles?. Spine J.

[61] Xu L, Chu B, Feng Y, Xu F, Zou Y-F (2016). Modic changes in lumbar spine: prevalence and distribution patterns of end plate oedema and end plate sclerosis. Br J Radiol.

[62] Dudli S, Liebenberg E, Magnitsky S, Miller S, Demir-Deviren S, Lotz JC (2016). *Propionibacterium acnes* infected intervertebral discs cause vertebral bone marrow lesions consistent with Modic changes. J Orthop Res.

[63] Dudli S, Miller S, Demir-Deviren S, Lotz JC (2018). Inflammatory response of disc cells against *Propionibacterium acnes* depends on the presence of lumbar Modic changes. Eur Spine J.

[64] Wedderkopp N, Thomsen K, Manniche C, Kolmos HJ, Secher Jensen T, Leboeuf Yde C (2009). No evidence for presence of bacteria in Modic type I changes. Acta Radiol.

[65] Albert HB, Sorensen JS, Christensen BS, Manniche C (2013). Antibiotic treatment in patients with chronic low back pain and vertebral bone edema (Modic type 1 changes): a double-blind randomized clinical controlled trial of efficacy. Eur Spine J.

[66] Braten LCH, Rolfsen MP (2019). Efficacy of antibiotic treatment in patients with chronic low back pain and Modic changes (the AIM study): double blind, randomised, placebo controlled, multicentre trial. BMJ.

[67] Palazzo C, Ferrari M, Lefevre-Colau MM, Nguyen C, Rannou F, Poiraudeau S (2017). Lack of effectiveness of antibiotics in chronic low back pain with Modic 1 changes. Joint Bone Spine.

[68] Gilligan CJ, Cohen SP, Fischetti VA, Hirsch JA, Czaplewski LG (2021). Chronic low back pain, bacterial infection and treatment with antibiotics. Spine J.

