# Supplementary material for: Evidence for infection in intervertebral disc degeneration: a systematic review
Source: Eur Spine J. 2021 Dec 4;31(2):414–30. doi: 10.1007/s00586-021-07062-1 (PMC8873132; doi:10.1007/s00586-021-07062-1)
Supplement: Supplementary file 1 — Supplementary file1 (DOCX 17 KB) [file 586_2021_7062_MOESM1_ESM.docx]

Supplementary Figure 1 (S1) Search strategy and results

MEDLINE: 273

PubMed: 52

Scopus: 60

Web of Science: 110

MEDLINE: (((Modic change OR intervertebral dis*)) AND ((bacteria OR microb*))) AND (infect*) Filters: from 2001 – 2021

PubMed: (((Modic change OR intervertebral dis*)) AND ((bacteria OR microb*))) AND (infect*) Filters: from 2001 – 2021

Scopus: (((Modic W/1 change OR intervertebral W/1 dis*)) AND ((bacteria OR microb*))) AND (infect*)

Web of Science: ((TS=(Modic change OR intervertebral dis*)) AND (TS=(bacteria OR microb*))) AND TS=(infect*) Filters: from 2001 – 2021

Two authors (IGS & PW) examined these results. 495 articles were found 151 duplicated removed leaving 344 articles assessed for inclusion in the review. 35 articles were identified for inclusion.

A ‘cited by’ reference search was conducted by one author (IGS) in PubMed on 05.04.21 to find any recent additional papers. One additional article was found in the ‘cited by’ lists of 6 included articles. A reference list search of all included articles was conducted by one author (PW) to check for any relevant papers. No additional, relevant articles were identified.

Google Scholar was searched as a secondary source by one author (IGS) on 04.04.21. Citations and patents were unchecked, with date limits of 2001- 2021 and the following search string was used:

"Modic change" OR "intervertebral disc" AND bacteria OR microbe AND infection

5,210 articles were returned and presented in the order of relevance. The first 15 pages (300 articles) were assessed for inclusion. One additional article was found.
